# Inpatient Psychiatry Placement Quality Improvement Project for Medical Students at University College London

**DOI:** 10.1192/bjo.2024.441

**Published:** 2024-08-01

**Authors:** Maja Swirska, Mustafa Abbas, Petros Lekkos, Suzanne Reeves, Stephen Ginn

**Affiliations:** 1 University College London Medical School, London, United Kingdom; 2 North London Mental Health Partnership, London, United Kingdom; 3 Central North West London NHS Trust, London, United Kingdom

## Abstract

**Aims:**

UCL 5th year medical students undertake 3-week North London Mental Health Partnership inpatient psychiatric wards placements.

Before this project the management of these placements was at the discretion of individual ward teams. A varied, and potentially unsatisfactory, medical student experience resulted.

This project sought to implement a structured approach to placements.

Ward teaching best practice was, for the purposes of this project, considered to be (i) sending students a welcome email prior to placement, (ii) issuing placement timetable, (iii) using tutorial materials for onward tuition.

Project aims: 75% of wards sending welcome email, 75% issuing timetables, 75% using tutorial materials, 75% of students stating placement exceeded expectations.

**Methods:**

This project consists of 1 PDSA cycle.

Prior to project baseline measures of ward teaching best practice were collected. The project started at the commencement of 2022/2023 academic year; duration: 12 weeks. The intervention was that inpatient medical teams were supported to send an introductory email to each student cohort, provide a placement timetable, and use supplied tutorial materials.

Questionnaires were emailed to inpatient medical teams at 6 and 12 weeks and to medical students at the end of placements. Medical team questionnaire covered engagement with best practice teaching. The student questionnaire addressed placement experience.

**Results:**

Outcomes at project conclusion:

- 33.3% of wards sent introductory email.
- 66.7% of wards issued a placement timetable.
- 16.7% of wards used tutorial materials.
- Less than 75% of student reported that the placement exceeded expectations.

Student experiences were varied: from excellent to feeling ignored. Students expressed a strong preference for additional structured teaching.

The medical inpatient teams did not engage with this project as hoped. Feedback suggested reasons:

- Lack of knowledge about the project.
- Time pressures.
- Perceived lack of medical student engagement.
- Team had preferred teaching practices.

**Conclusion:**

Despite this intervention, student inpatient placement experience remains varied.

It may have been optimistic to expect medical teams to change their established practice regarding medical students with only very modest additional support.

Some teams are enthusiastic and thoughtful about student teaching. Other are less so; this may be associated with temporary staff.

Following PDSA cycle 1 no further cycles were attempted as outcome suggested an alternative approach is required.

Possibilities for further PSDA cycles include:

- Supporting placements via regular teaching-focused ward team meetings where expertise can be shared.
- Appointing ward teaching fellows.
- Explicitly rewarding inpatient teams displaying teaching excellence.

